# Impact of Atrial Fibrillation on Patients With Inflammatory Bowel Disease Admitted for Colectomy

**DOI:** 10.7759/cureus.27849

**Published:** 2022-08-10

**Authors:** Ratib Mahfouz, Mustafa F Douglas, Adham E Obeidat, Mohammad Darweesh, Mahmoud M Mansour, Parthav Shah, Mohammad Aldiabat, Yazan Aljabiri, Angela Fishman

**Affiliations:** 1 Internal Medicine, Kent Hospital/Brown University, Warwick, USA; 2 Internal Medicine, Midwestern University Arizona College of Osteopathic Medicine, Sierra Vista, USA; 3 Internal Medicine, University of Hawaii, Honolulu, USA; 4 Internal Medicine, East Tennessee State University, Johnson City, USA; 5 Internal Medicine, University of Missouri School of Medicine, Columbia, USA; 6 Internal Medicine, Hawaii Residency Program, Honolulu, USA; 7 Internal Medicine, Lincoln Medical Center, New York, USA; 8 Internal Medicine, Lincoln Medical Center, Bronx, USA; 9 Gastroenterology, Kent Hospital/Brown Unviersity, Warwick, USA

**Keywords:** inpatient, worse outcome, colectomy, ibd, atrial fibrillation

## Abstract

Introduction

Inflammatory bowel disease (IBD) is a chronic, relapsing, inflammatory disorder of the gastrointestinal tract. Patients with IBD may undergo a segmental or total colectomy, depending upon the extent of the disease. It is estimated that approximately 20 to 30 percent of patients with advanced ulcerative colitis will eventually require surgical resection. The incidence and prevalence of Atrial Fibrillation (AF) are increasing globally. There is plausible evidence linking inflammation to the initiation and perpetuation of AF. Given the importance of systemic inflammation in the pathogenesis of AF, an increased risk of the development of other diseases related to systemic inflammation can be expected.

Objective

Study how AF can affect the outcome of the patients in a population database hospitalized due to IBD flare and in whom colectomy was performed.

Methodology

Data from the National Inpatient Sample database from 2016 to 2019 were used to obtain baseline demographic numbers and outcome variables. T-tests and chi-square tests were used to compare data. Univariate and multivariate logistic regression was used to calculate Odds ratios for comorbidities.

Results

The study identified 27,165 patients with IBD who had colectomy during the same admission, among whom 2,045 also had AF. AF patients had a statistically significant longer mean LOS than patients without AF (16.79 vs. 11.24 days, p-value 0.001). AF patients also had significantly higher hospital charges ($222,109 vs. $142,011, p-value < 0.001). The mortality rate in IBD undergoing colectomy patients with AF was higher than in patients without AF (13.45% vs. 2.69%, p-value < 0.001), which was also reflected in multivariate analysis with an odds ratio of 2.27 (p-value < 0.001) after adjusting for age, gender, race, and comorbidities.

Conclusion

Our study showed that a national cohort of IBD patients with a history of colectomy had increased mortality and morbidity in the presence of AF. A finding that can guide physicians to allocate more time to optimizing the management of AF in this group of patients decreases the risk of complications, length of stay, and overall mortality.

## Introduction

Inflammatory bowel disease (IBD) is a chronic, relapsing, inflammatory disorder of the gastrointestinal tract. It includes ulcerative colitis (UC) and Crohn’s disease (CD), which show differences in pathology and clinical characteristics [1]. The incidence and prevalence of IBD are increasing worldwide, indicating its emergence as a global disease [2].

Patients with CD and UC may undergo a segmental or total colectomy, depending upon the extent of the disease. Treatment choices should be individualized based on patient characteristics, preferences, and available resources [3]. It is estimated that approximately 20 to 30 percent of patients with advanced ulcerative colitis will eventually require surgical resection [4]. 

Atrial Fibrillation (AF) incidence and prevalence are on the rise around the world. The prevalence of AF has increased threefold over the last 50 years, according to the Framingham Heart Study. [5] At least 3 to 6 million people in the united states alone have AF, these numbers are projected to reach up to 16 million by 2050 [6]. Various inflammatory markers such as C-reactive protein, tumor necrosis factor-α, and interleukin-2, 6, and 8 have been associated with AF. There is plausible evidence linking inflammation to the initiation and perpetuation of AF [7].

Given the importance of systemic inflammation in the pathogenesis of AF, an increased risk of developing other diseases related to systemic inflammation can be expected. In this article, we will study how AF can affect the outcome of the patients in a population database hospitalized due to IBD flare and in whom the colectomy was performed.

## Materials and methods

Data source

We conducted a retrospective cohort study for patients admitted to hospitals with a primary diagnosis of IBD who underwent colectomy in the United States from 2016 to 2019 from the database of Healthcare Cost and Utilization Project National Inpatient Sample (NIS). This database is the largest publicly available inpatient health care database in the USA, which sponsored by the Agency for Healthcare Research Quality (AHRQ). This database covers more than 97% of the US population [8].To ensure national representation, a 20 percent probability sample was collected and subsequently weighted. The variables are defined via the International Classification of Disease, 10th revision, and Clinical Modification (ICD-10-CM) codes.

Study variables

Patients younger than 18 years old were excluded. Patient’s age (in years), gender, race (White, Black, Hispanic, Others), and hospital information (region and bed size) were collected and considered as baseline characteristics. Using ICD-10-CM codes, we were able to identify patients who carry certain comorbidities, including AF, hypertension (HTN), diabetes mellitus (DM), congestive heart disease (CHF), chronic kidney disease (CKD), chronic obstructive pulmonary disease (COPD), and coronary artery disease (CAD). Outcome data, such as total hospital charge, length of stay, in-hospital mortality, and requiring mechanical ventilation were also collected.

Statistical analysis

STATA software, version 17.0 (StataCorp., College Station, TX, USA), was used for the statistical analysis. Descriptive statistics, such as t-test, and chi-square were used to describe the characteristics of patients with IBD undergoing colectomy with and without AF. We used univariate and multivariate logistic regression analyses to determine factors associated with the studied outcomes. We excluded variables that were not statistically significant (p-value > 0.05) in univariate analysis from the multivariate analysis. For the study and outcome variables, we used odds ratios at 95% confidence intervals. Two-tailed p-values of 0.05 or lower were considered statistically significant.

## Results

Patients and hospital characteristics

**Figure 1:**
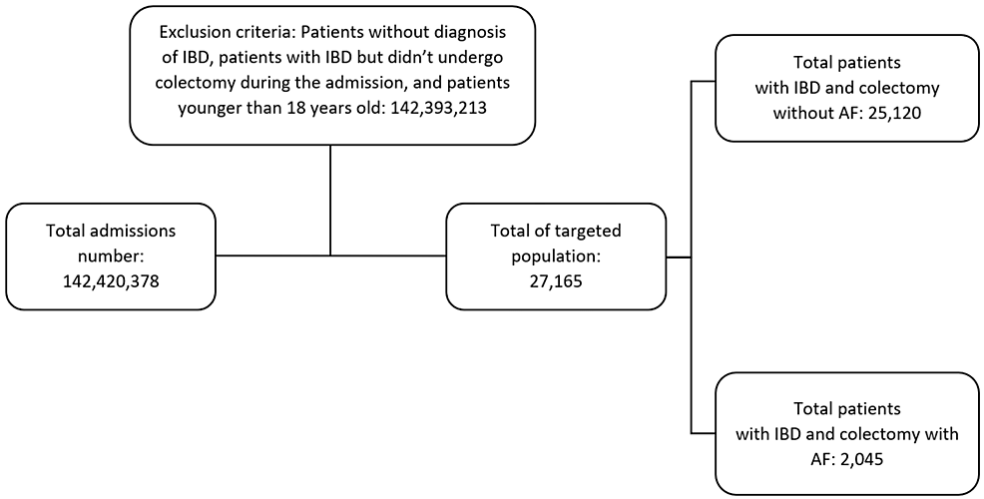
Population's selection criteria for patients with IBD undergoing colectomy with or without atrial fibrillation IBD: Inflammatory bowel disease; AF: Atrial fibrillation

We identified 27,165 patients with IBD undergoing colectomy during the same admission, among which 2,045 patients also had AF diagnoses (Figure 1).

Patients with AF were significantly older than patients without AF (mean of 71 vs. 49 years, P<0.001). Gender distribution was nearly identical in the no AF group to the AF group, while the male gender was more prevalent in the AF group (54.77%). The white race was the most prevalent in both groups but more common in the AF group (77.59% vs. 85.33%; p-value 0.0013). The most common hospital characteristic is a large hospital in the southern region. The comorbidities were more prevalent in the AF group (Table 1).

**Table 1:** Demographic and clinical characteristics of patients with and without atrial fibrillation IBD: inflammatory bowel disease; AF: atrial fibrillation; HTN: hypertension; DM: diabetes mellitus; CHF: congestive heart disease; CKD: chronic kidney disease; COPD: chronic obstructive pulmonary disease; CAD: coronary artery disease.

Variable	No AF	AF	P-value
Age (mean, yr)	49	71	<0.001
Gender (%)			0.0271
Male	49.15%	54.77%	
Female	50.85%	45.23%	
Race (%)			0.0013
White	77.59%	85.33%	
Black	9.85%	5.38%	
Hispanic	5.23%	4.65%	
Others	7.32%	5.65%	
Hospital region (%)			0.4263
Northeast	21.68%	23.72%	
Midwest	24.82%	24.94%	
South	37.08%	37.9%	
West	16.42%	13.45%	
Hospital bed size (%)			0.0005
Small	13.59%	16.14%	
Medium	24.64%	31.78%	
Large	61.76%	52.08%	
Comorbidities (%)			
HTN	30.91%	66.26%	<0.001
DM	10.59%	23.72%	<0.001
CHF	3.26%	24.45%	<0.001
CKD	5.65%	21.52%	<0.001
COPD	6.47%	19.56%	<0.001
CAD	7.36%	30.56%	<0.001
Smoking	35.69%	36.43%	0.7636
Obesity	11.96%	17.11%	0.0016

Inpatient outcomes

Length of Stay and Total Hospital Charges

The mean length of stay (LOS) was statistically significantly longer than in patients with AF compared to patients without AF (16.79 vs. 11.24 days, p-value < 0.001). Total hospital charges were also significantly higher in AF group ($222,109 vs. $142,011, p-value < 0.001). Table 2 summarizes these findings.

Mortality

**Figure 2:**
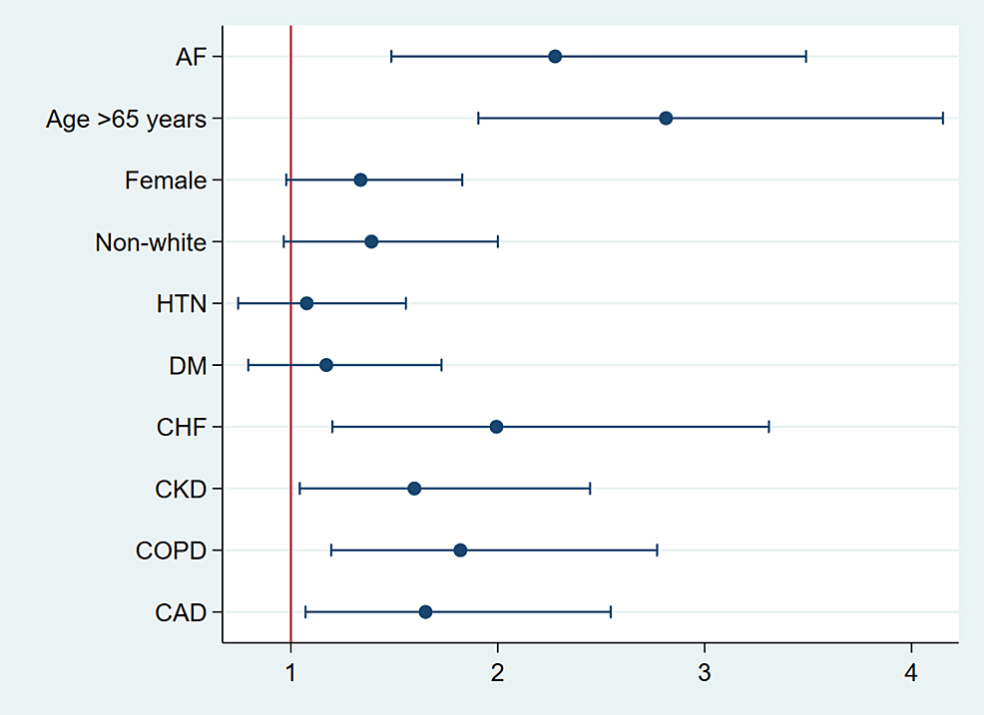
Odds ratio plot of mortality in patients with IBD who underwent colectomy IBD: inflammatory bowel disease; AF: atrial fibrillation; DM: diabetes mellitus; CHF: congestive heart disease; CKD: chronic kidney disease; COPD: chronic obstructive pulmonary disease; CAD: coronary artery disease.

Mortality rate in IBD undergoing colectomy patients with AF was higher than in patients without AF (13.45% vs. 2.69%, p-value < 0.001) (Table 2), which was also reflected in multivariate analysis with an odds ratio of 2.27 (p-value < 0.001) after adjusting for age, gender, race, and comorbidities. Patients older than 65 years old had an odds ratio of 2.81 of dying during hospitalization (p-value < 0.001). CHF, COPD, CKD, then CAD were statistically significant risk factors to increase in-hospital mortality. Gender, race, obesity, smoking, and race were not statistically significant (Table 3). A plot summarizes the results shown in Figure 2.

**Table 2:** Comparison of outcomes between patients with IBD undergoing colectomy with AF vs without AF IBD: inflammatory bowel disease; AF: atrial fibrillation; USD: United States Dollar.

Outcome	No AF	AF	P-value
Total hospital charge ($)	142,011$	222,109$	<0.001
LOS (days)	11.24	16.79	<0.001
Death (%)	2.69%	13.45%	<0.001
Ventilation	7.21%	22.25%	<0.001
Cardiac arrest	1.13%	6.11%	<0.001
ICU admission	8.46%	23.47%	<0.001

**Table 3:** Odds ratio table for predictors of mortality in IBD patients undergoing colectomy IBD: inflammatory bowel disease; AF: atrial fibrillation; HTN: hypertension; DM: diabetes mellitus; CHF: congestive heart disease; CKD: chronic kidney disease; COPD: chronic obstructive pulmonary disease; CAD: coronary artery disease.

Died	OR (CI 95%)	P-value	aOR (CI 95%)	P-value
AF	5.62 (4-7.86)	<0.001	2.27 (1.48-3.49)	<0.001
Age <65 years	-	-	-	-
Age ≥ 65 years	5.03 (3.71-6.82)	<0.001	2.81 (1.9-4.15)	<0.001
Male	-	-	-	-
Female	1.36 (1.01-1.83)	0.037	1.33 (0.97-1.82)	0.069
White	-	-	-	-
Non-white	1.02 (0.72-1.44)	0.919	1.38 (0.077-2)	0.077
HTN	2.64 (1.98-3.52)	<0.001	1.07 (0.74-1.55)	0.694
DM	2.32 (1.63-3.29)	<0.001	1.17 (0.79-1.72)	0.425
CHF	5.97 (4.09-8.69)	<0.001	1.99 (1.2—3.31)	0.008
CKD	3.94 (2.74-5.65)	<0.001	1.59 (1.2-3.31)	0.031
COPD	3.81 (2.66-5.45)	<0.001	1.81 (1.19-2.77)	0.005
CAD	4.44 (3.21-6.15)	<0.001	1.65 (1.07-2.54)	0.023
Smoking	0.8 (0.58-1.09)	0.171	-	-
Obesity	1.29 (0.86-1.93)	0.217	-	-

Mechanical Ventilation

Univariate and multivariate analysis demonstrated that AF remains a major contributor for mechanical ventilation (aOR 2.05, CI 95% 1.48-2.84, p-value < 0.001) in the studied population. Age ≥ 65 years, female gender, non-white race, diabetes, CKD, COPD and CAD were also found to increase odds ratio for mortality (Table 4).

**Table 4:** Odds ratio table for predictors of mechanical ventilation in IBD patients undergoing colectomy IBD: inflammatory bowel disease; AF: atrial fibrillation; HTN: hypertension; DM: diabetes mellitus; CHF: congestive heart disease; CKD: chronic kidney disease; COPD: chronic obstructive pulmonary disease; CAD: coronary artery disease.

Mech. Ventilation	OR (CI 95%)	P-value	aOR (CI 95%)	P-value
AF	3.68 (2.84-4.77)	<0.001	2.05 (1.48-2.84)	<0.001
Age <65 years	-	-	-	-
Age ≥ 65 years	2.58 (2.11-3.15)	<0.001	1.59 (1.22-2.07)	0.001
Male	-	-	-	-
Female	1.38 (1.14-1.68)	<0.001	1.37 (1.12-1.68)	0.002
White	-	-	-	-
Non-white	1.07 (0.85-1.34)	0.544	1.3 (1.03-1.65)	0.027
HTN	1.83 (1.51-2.22)	<0.001	0.96 (0.75-1.23)	0.776
DM	2.1 (1.63-2.17)	<0.001	1.34 (1-1.8)	0.004
CHF	3.57 (2.64-4.83)	<0.001	1.47 (0.99-2.17)	0.053
CKD	3.25 (2.46-4.28)	<0.001	1.81 (1.31-2.51)	<0.001
COPD	3.18 (2.45-4.13)	<0.001	2.01 (1.48-2.72)	<0.001
CAD	2.94 (2.29-3.77)	<0.001	1.46 (1.07-1.99)	0.015
Smoking	0.87 (0.7-1.07)	0.191	-	-
Obesity	1.33 (1.01-1.74)	0.037	1.16 (0.86-1.54)	0.313

Cardiac Arrest

AF was found to have an increased risk for cardiac arrest (aOR 3.3, CI 95% 1.69-6.46, p-value < 0.001). Age ≥ 65 years and CAD were the only other studied variables were found to increase odds ratio for mortality (Table 5).

**Table 5:** Odds ratio table for predictors of cardiac arrest in IBD patients undergoing colectomy IBD: inflammatory bowel disease; AF: atrial fibrillation; HTN: hypertension; DM: diabetes mellitus; CHF: congestive heart disease; CKD: chronic kidney disease; COPD: chronic obstructive pulmonary disease; CAD: coronary artery disease.

Cardiac arrest	OR (CI 95%)	P-value	aOR (CI 95%)	P-value
AF	5.67 (3.5-9.17)	<0.001	3.3 (1.69-6.46)	<0.001
Age <65 years	-	-	-	-
Age ≥ 65 years	2.89 (1.86-4.49)	<0.001	1.72 (1-2.98)	0.049
Male	-	-	-	-
Female	0.98 (0.63-1.51)	0.938	1.02 (0.65-1.59)	0.912
White	-	-	-	-
Non-white	1.15 (0.69-1.92)	0.571	1.41 (0.84-2.37)	0.185
HTN	1.81 (1.17-2.8)	0.008	0.89 (0.53-1.49)	0.672
DM	1.06 (0.54-0.206)	0.86	-	-
CHF	3.82 (2.8-7)	<0.001	1.34 (0.56-3.18)	0.5
CKD	2.87 (1.6-5.13)	<0.001	1.37 (0.69-2.7)	0.357
COPD	1.54 (0.76-3.1)	0.225	-	-
CAD	4.02 (2.46-6.59)	<0.001	2.14 (1.2-3.84)	0.01
Smoking	1.09 (0.69-1.71)	0.695	-	-
Obesity	1.1 (0.58-2.08)	0.768	-	-

ICU Admission

AF was found to have an increased of odds ratio for ICU admission (aOR 1.87, CI 95% 1.37-2.56, p-value < 0.001). Age ≥ 65 years, female gender, CKD, COPD, and CAD were also found to have an increased odds ratio (Table 6).

**Table 6:** Odds ratio table for predictors for ICU admission in IBD patients undergoing colectomy IBD: inflammatory bowel disease; AF: atrial fibrillation; HTN: hypertension; DM: diabetes mellitus; CHF: congestive heart disease; CKD: chronic kidney disease; COPD: chronic obstructive pulmonary disease; CAD: coronary artery disease.

ICU admission	OR (CI 95%)	P-value	aOR (CI 95%)	P-value
AF	3.31 (2.57-4.27)	<0.001	1.87 (1.37-2.56)	<0.001
Age <65 years	-	-	-	-
Age ≥ 65 years	2.48 (2.05-2.98)	<0.001	1.57 (1.23-2)	<0.001
Male	-	-	-	-
Female	1.38 (1.15-1.66)	<0.001	1.38 (1.14-1.67)	0.001
White	-	-	-	-
Non-white	1.04 (0.83-1.29)	0.721	1.24 (0.99-1.57)	0.058
HTN	1.77 (1.48-2.13)	<0.001	0.98 (0.78-1.24)	0.903
DM	1.93 (1.52-2.47)	<0.001	1.27 (0.96-1.67)	0.087
CHF	3.37 (2.51-4.53)	<0.001	1.49 (1.02-2.16)	0.0
CKD	3.36 (2.58-4.39)	<0.001	1.99 (1.46-2.72)	<0.001
COPD	2.99 (2.33-3.84)	<0.001	1.91 (1.43-2.55)	<0.001
CAD	2.67 (2.09-3.41)	<0.001	1.35 (1-1.81)	0.047
Smoking	0.97 (0.72-1.06)	0.176	-	-
Obesity	1.21 (0.93-1.58)	0.14	-	-

## Discussion

Our results are concordant compared to a similarly designed study that examined AF in patients with IBD [9]. However, this retrospective study is the first to evaluate the effect of AF on outcomes in hospitalized patients with IBD undergoing colectomy. Furthermore, in addition to mortality risk, we also studied the risk of requiring mechanical ventilation, developing cardiac arrest, and ICU admission.

The primary outcome of our study is that there is an increased risk of death in IBD patients who underwent colectomy if they also had AF compared to those without arrhythmia. We also found that AF in those patients is an independent risk factor that increases the risk of cardiac arrest, mechanical ventilation, and ICU admission. Overall, the total hospital charge and length of stay were markedly increased in the hospitalized IBD patients with colectomy who also were comorbid with AF. 

AF, the most common sustained cardiac arrhythmia, is becoming more prevalent in an aging population [6]. The prevalence of AF among our targeted population is significantly higher than the prevalence of AF in the general US population (7.52% vs. 0.4-1%) [10]. The pathophysiology of AF has been well studied and has been more understood over the years [11]. The inflammation's role in developing AF has been demonstrated by analyzing the relationship between the elevation of CRP levels and the presence of AF [12]. Whether initiation of AF activates direct inflammatory effects or whether the presence of a preexisting systemic inflammatory state promotes further persistence of AF remains unclear. 

Several proinflammatory and immune-regulatory cytokines are upregulated in the mucosa of patients with IBD and demonstrate the disease's inflammatory nature [13]. CRP is the most widely used serum indicator of inflammation in IBD. Increased levels of CRP help differentiate active mucosal disease from quiescent IBD. CRP level <10 mg/l indicates the remission stage of IBD [14]. 

Data suggest that thrombosis is a specific feature of IBD that can be involved in thromboembolic events and the pathogenesis of the disease. The data showed that patients with IBD, both CD, and UC, are at an increased risk for arterial thromboembolism, mainly cerebral vascular disease, ischemic heart disease, and mesenteric ischemia [15]. Evidence from the literature suggests that thrombosis is a specific feature of IBD involved in thromboembolic events and the pathogenesis of the disease itself [16]. Thromboembolism is the most important complication of AF, and AF is the most common factor in stroke in the elderly. The determinants of the Virchow triad, including stasis, endothelial damage, and coagulation properties, are centrally involved in AF-related thrombus formation [17].

Pathophysiological similarities between the two entities can explain the co-occurrence of AF and IBD; namely, inflammation demonstrated with elevated CRP levels and the increased risk of thromboembolic events in both diseases. The basis of the significant outcome of this study which is increased mortality in IBD patients who underwent colectomy if they were comorbid with AF, can be attributed in part to the inflammatory nature of both diseases; subsequently, monitoring the inflammatory markers of AF can give additional insight about the prognosis for patients with IBD. This national-level study shows the strong association of worse outcomes for IBD patients who underwent colectomy with AF can direct new management approaches for hospitalized patients with IBD. 

Limitations

Firstly, The NIS database uses ICD-10 CM codes for disease diagnoses that may be subject to error. Secondly, this database based on in-patient discharges, and each admission is considered as an independent event even if it was for the same patient who was admitted multiple times. And lastly, this study is an observational study, and unmeasured confounding factors may influence these findings.

## Conclusions

To conclude, systemic inflammation can be linked to the development and persistence of AF which can explain the increased incidence of AF in inflammatory bowel disease patients. Mortality was higher in IBD patients who are undergoing colectomy with AF compared to those patients without AF. The presence of AF was an independent risk factor that increased the risk of cardiac arrest, mechanical ventilation, length of stay, and ICU admission. We believe that those findings can guide physicians to allocate more time for optimizing the management of AF in this group of patients to decrease the risk of complications, length of stay, and overall mortality.
